# Topology-commanded optical properties of bistable electric-field-induced torons in cholesteric bubble domains

**DOI:** 10.1038/s41598-017-16241-4

**Published:** 2017-11-23

**Authors:** Andrii Varanytsia, Gregor Posnjak, Urban Mur, Vinay Joshi, Kelsey Darrah, Igor Muševič, Simon Čopar, Liang-Chy Chien

**Affiliations:** 1 Liquid Crystal Institute, Kent State University, Kent, Ohio, USA; 2 Condensed Matter Physics Department, Jožef Stefan Institute, Ljubljana, Slovenia; 3 Faculty of Mathematics and Physics, University of Ljubljana, Ljubljana, Slovenia

## Abstract

Nowadays, complicated topological defects enable many experimental manipulations and configurational simulations of active soft matter for optical and photonic applications. Investigation of topological defects in soft anisotropic materials enables one to better understand three-dimensional orientation fields in cholesteric liquid crystals. Here, we describe optical properties of bistable bubble domain (BD) texture torons in a thin layer of cholesteric liquid crystal (CLC), frustrated by homeotropic anchoring conditions, and reliably switchable by a random process. The control of macroscopic optical density and diffraction efficiency of the BD texture is demonstrated by a selection of a confinement ratio of the CLC. Experimentally reconstructed CLC director profile reveals the topology of BD torons allowing consideration of naturally occurring BD texture for applications in optical and photonic devices, which are bistably switchable between active and transparent optical states.

## Introduction

Liquid crystals (LC) are extraordinary materials that stand out uniquely among numerous types of periodic dielectric materials for photonic applications. LCs are soft and they respond strongly to external stimuli, such as electric and magnetic fields, they are able to adapt and self-assemble into a variety of structures that exhibit new photonic phenomena^1^. Moreover, the collective orientational order of LCs gives rise to numerous types of topological defects, which transfer topological singularities to light and could be exploited in novel devices based on singular photonics. Control and understanding of the nature of topological defects in liquid crystals is nowadays a topic of outmost interest.

Cholesteric liquid crystals (CLC) are particularly rich in structural diversity of topological defects and have a great potential for future use in novel photonic devices. The CLCs  have a supermolecular helical structure characterized by a helical pitch *p*_0_ over which the CLC director rotates by 2π. By controlling the amount of chiral additives in the CLC mixture the helical pitch can be selected to be comparable to the wavelength of light enabling unique optical and photonic properties from UV to IR range of spectrum.

When a CLC is confined between surfaces treated for homeotropic alignment the geometric frustration deforms the CLC helix in order to satisfy the surface boundary conditions resulting in formation of distinct LC textures. The appearance of CLC textures with such confinement depends on the dimensionless confinement ratio of the cell gap thickness *d* over the equilibrium CLC pitch *p*_0_: *C* = *d*/*p*_0_. At small values of confinement ratio the helical structure of the CLC is suppressed by anchoring force of the alignment layer and CLC director is unwound into a uniform homeotropic (HO) texture. At large confinement ratios CLC forms a fingerprint (FP) texture consisting of a series of periodic striped domains, which are often parallel to each other. The LC director in a FP texture fulfills homeotropic orientation boundary conditions next to confining surfaces, but is twisted in the bulk of the CLC layer^2,3^. The FP texture forms spontaneously if the confinement ratio is larger than a critical value, *C*_c_. The *C*_c_ corresponds to a cell gap thickness at which competing surface anchoring force and elastic force twisting the CLC director into a periodic helical structure are naturally balanced^4,5^.

The CLCs with the confinement ratio in the vicinity of the *C*_c_ are known to form a LC texture which is distinct from the FP and HO textures and is historically called a bubble domain (BD) texture^5–7^. After initial reports substantial work had been done to understand the properties of the BD texture^8^, the mechanism of its formation^9–11^, and topology of the CLC director inside of the bubbles^10,12–16^. The BD texture consists of a single layer of uniformly sized three-dimensional inhomogeneous configurations of СLC director, which are embedded into a uniform homeotropic background.

The BD texture can be generated in a homeotropic cell using AC electric field with a CLC with negative dielectric anisotropy (Δ*ε* < 0)^5–7,11,12,17,18^. At sufficient magnitude the electric field significantly affects molecular alignment by electro-hydrodynamic forces arising from electrical conduction in the LC layer during which the momentum of motion of free charges is transferred through viscous friction to LC molecules. This hydrodynamic instability causes the cell to appear bright due to dynamic scattering (DS) of light on the fluctuating director. Some of topological defects generated by random turbulent flow after the onset of DS become confined into a metastable director configuration forming the BD texture after the applied electric field is removed. СLC with positive dielectric anisotropy (Δ*ε* > 0) can generate BD texture using a rapid isotropic to chiral nematic phase transition during quenching of the CLC layer^5,8,10,12^.

Topologically nontrivial structure and properties of metastable particle-like excitations of CLC director with an equivalent confinement called torons and skyrmions have been the subject of thorough recent explorations^19–29^. Torons were generated and manipulated optically with vortex or unstructured^20,22,30–35^ laser beams focused on the CLC layer, modified by colloidal inclusions^36–38^, observed in the microchannel^39,40^ and droplet geometry^41^. The BD torons behave as particle-like entities with short range repulsive interactions without external fields and dipole-like attractive interactions arising from electrostriction in a weak external electric field^11,42^. Analogous to other 2D colloidal systems with soft shoulder repulsion^43^, they demonstrate the ability to self-assemble into hexagonally ordered domains allowing their consideration for potential applications in diffractive optical elements and bistable light shutters^17,18,35,44,45^. The topology and mutual interactions of torons and other metastable excitations of the CLC director are demonstrated to be significant to a wide variety of topics within and beyond the soft mater physics^21,24,27,28,46^.

Regardless the significant previous scientific interest the macroscopic optical properties of CLC BD texture have only been reported describing the diffraction of monochromatic laser light^5,12,17,44^, or transmittance as a function of the wavelength of light at a fixed confinement ratio^45^. In this work, we discuss the dependence of macroscopic transmittance and diffraction efficiency of electric-field-induced BD texture on the values of the confinement ratio and size of bubble domains, and demonstrate an experimentally reconstructed 3D CLC director structure of a BD toron. Our findings provide a potential approach for the control of macroscopic optical properties of a CLC layer containing spontaneously formed BD torons, and enable explicit comparison of BD torons with other members of the family of metastable excitations of CLC director.

## Experimental Results and Discussion

**Figure 1:**
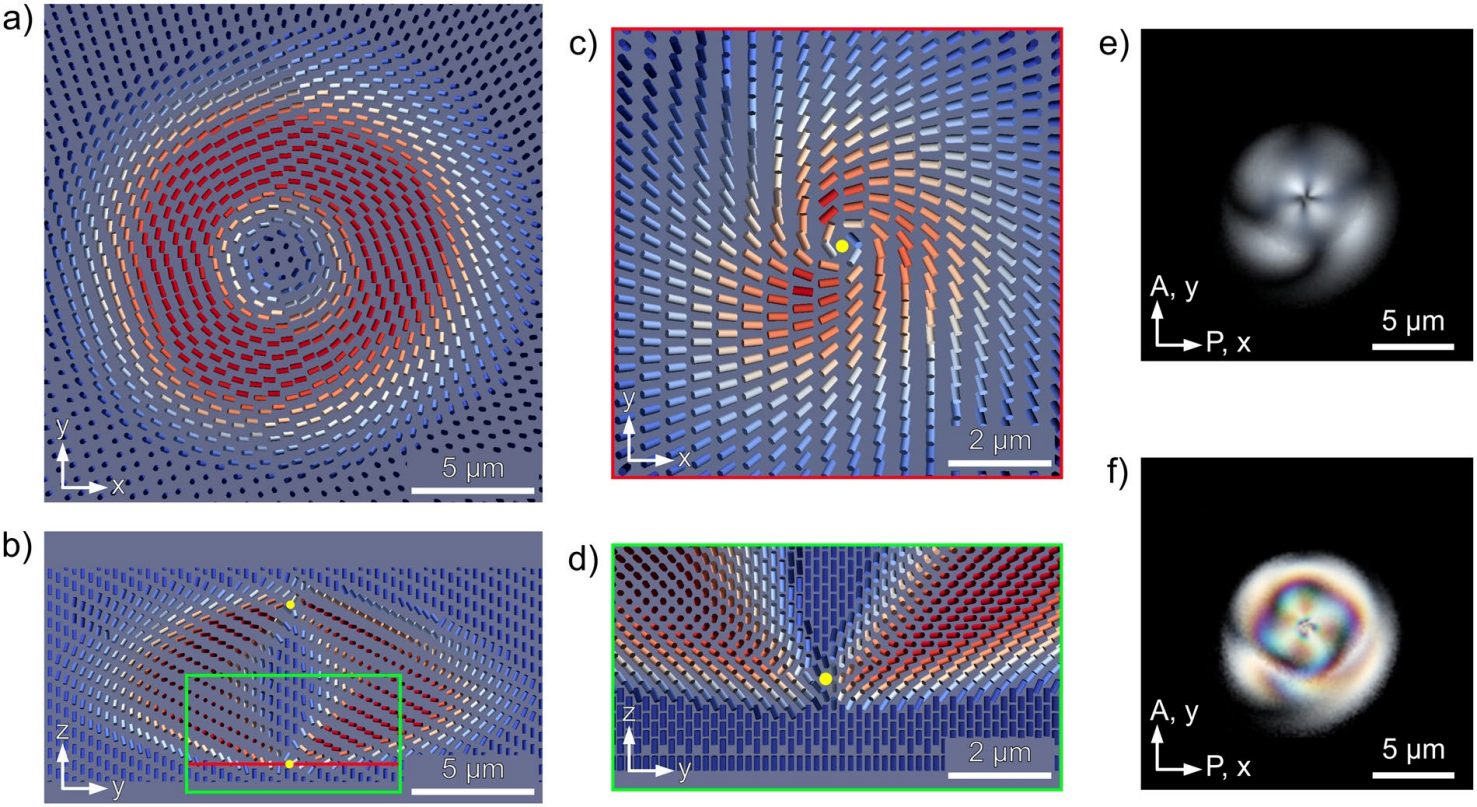
The cross-section views of FCPM reconstructed 3D LC director configuration of a spontaneously formed BD toron in: (**a**) horizontal midplane perpendicular to the axis of the toron, and (**b**) vertical plane dissecting the center of the toron. Close-up cross-section views dissecting the bottom point defect lying on toron’s central axis in: (**c**) horizontal plane, and (**d**) vertical plane. The cylinders representing the director are colored by the size of their projection on the horizontal plane. The details of sharp director features such as point defects may be smeared in the reconstructed director field by the inherent LC fluctuations. The yellow dots mark the approximate positions of defects. The POM textures calculated from reconstructed 3D director structure using: (**e**) low birefringence, Δn = 0.03, and (**f**) high birefringence, Δn = 0.18.

The LC director configuration of a naturally occurring toron of the BD texture is experimentally reconstructed using fluorescence confocal polarizing microscopy (FCPM) imaging technique^41,47^. The BD texture torons for FCPM imaging were formed by quenching the LC cell from the isotropic phase to the LC phase at room temperature. Figure 1 shows cross-section views of a 3D LC director structure of a single standing BD toron. The local LC director is represented with cylinders, colored by the size of their projection on the plane of substrate of the LC cell (horizontal plane), and rendered in ParaView software. The cross-section views are in the horizontal midplane of LC layer parallel to the cell substrates (Fig. 1a), and in the vertical plane dissecting the BD toron through its center (Fig. 1b). The vertical plane cross-section shows that BD torons are metastable excitations of CLC director formed by a double twist cylinder bent into a torus-like shape, which is positioned between two localized defect structures – two topological point defects. The close-up view of a point defect shows that the director is radial but twisted in a horizontal cross-section which dissects the defect and oriented azimuthally close to the defect as seen in Fig. 1c. In the vertical cross-section (Fig. 1d) the defect has a hyperbolic profile. The structure of the top and bottom point defects in the reconstructed BD torons agrees with the experimental and numerical data of both laser induced^20^ and naturally occurring T3-1 torons^42^ and is similar to the structure of point defects found close to the homeotropic surface of cholesteric droplets^41,48^.

The 3D LC director structure obtained by FCPM reconstruction is used to calculate the polarizing optical microscopy (POM) texture of a FCPM imaged BD toron, which enables us to compare it with the texture of its directly observed counterparts. The POM images were calculated using a Jones matrix method generalized to account for the oblique propagation of light rays, enabling the reproduction of optical effects of light focusing by the microscope lens with a large numerical aperture^49^. The POM image calculated using a low birefringence value (Fig. 1e) shows clearly visible four bright quarters in the center of the toron similarly to the smallest BD torons observed in our experiments, and closely resembling the naturally occurring BD torons in low birefringence CLC mixture^42^ or metastable skyrmions in thin CLC layer with an equivalent confinement^40^. However, phase retardation from larger birefringence or thicker CLC layers significantly changes the POM appearance of a toron, making direct visual comparison less straightforward, as shown in Fig. 1f. A notable difference in appearance between the simulated and the experimental POM images is mainly caused by the lensing effects of BD torons which are not taken into account by the simulation, and additionally by small movement of torons and LC director fluctuations obscuring the alignment of FCPM images taken at different polarizations of light.

**Figure 2:**
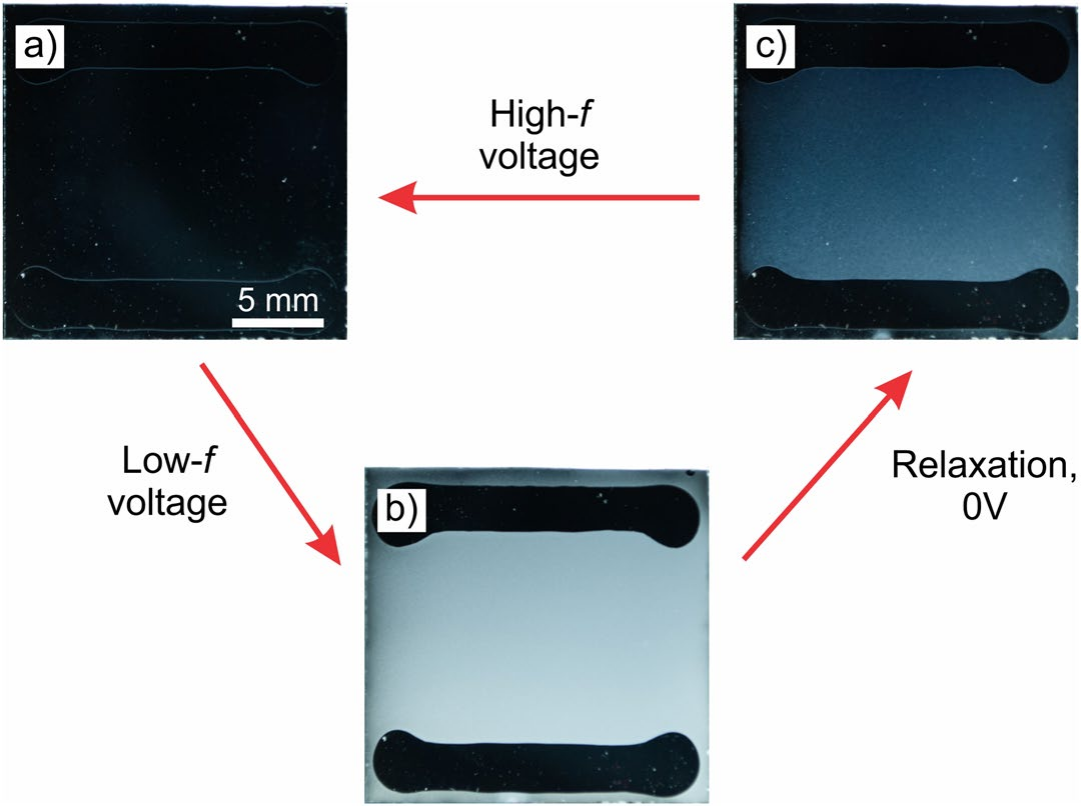
Unpolarized light photographs of a sample in front of black background switched between: (**a**) transparent HO texture at 0 V, (**b**) active DS state at 2.9 V/μm, 100 Hz, and (**c**) multidomain diffractive BD texture at 0 V. Cell gap is 7.0 µm and the confinement ratio is *C* = 0.68.

The BD texture for electro-optic experiments was generated with a low-frequency (100 Hz) AC electric field and empirically optimized amplitude in the range from ~1.5 V/µm in 24 μm-thick cells to ~6.5 V/µm in 3 μm-thick cells to yield the largest density of BD torons, and erased by a high-frequency (5 kHz) AC electric field of the same amplitude. The appearance of the sample in the DS state during the switching between HO and BD textures is shown in Fig. 2. Switching between HO (Fig. 2a) or FP and BD (Fig. 2c) texture consists of two steps^17^. First is the initiation of low-frequency electric-field-induced DS (Fig. 2b) or high-frequency induced translationally invariant configuration (TIC) states with typical characteristic response time in the range from 20 ms to 50 ms. Second, is a slower relaxation of distorted CLC director back to an equilibrium HO, FP, or metastable BD textures with a typical characteristic relaxation time in the range from 0.4 s to 2.1 s. Exact response and equilibrium times depend on the confinement ratio, the cell gap thickness of a sample, and viscoelastic properties of the CLC mixture.

The BDs form during the AC electric-field-induced hydrodynamic instability in the DS regime, where defects can form because of the turbulence. After the external electric field is removed the LC director relaxes locking some of the defects into a self-compensating topologically protected configurations of twisted bubble domain toron structures, as presented in Fig. 1. A balance between the elastic force of the deformed LC director and the anchoring force acting on the LC by the alignment layer creates an elastic energy barrier stabilizing the topological defects within the BD torons^20^. Because the torus-like double twist cylinder of the toron cannot be removed by smooth relaxation of the director field^20,23^, the BD torons behave as isolated topological objects metastable to HO or FP textures which can be formed at the same confinement ratio.

The erasing of the BD texture is performed by going through the translationally invariant configuration (TIC) texture. At the higher AC field frequency the ions and the LC molecules cannot follow the modulation of the electric field and due to Fréedericksz transition and negative dielectric anisotropy the LC molecules tilt away from the electric field direction^50^. The chirality of the LC causes the direction of tilted molecules to rotate around the direction of the electric field producing the TIC state. The TIC state is stable either under applied electric field or at larger confinement ratios than the BD texture, and it erases the torus-like central part of the torons, enabling continuous relaxation to the HO or FP texture. The relaxation happens after the high-frequency AC electric field is turned off because the studied confinement ratios are too small to support a stable TIC structure.

**Figure 3:**
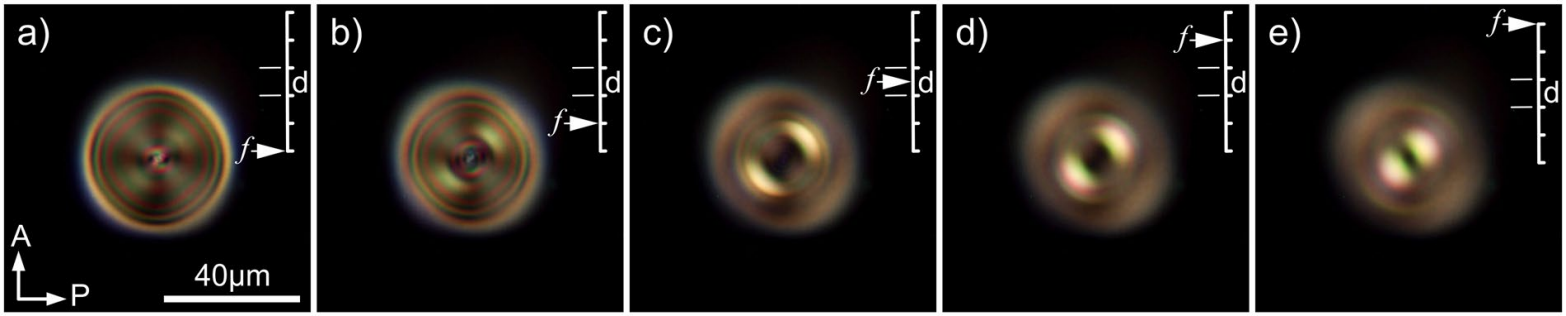
POM micrographs of a single BD toron taken with focusing of a microscope camera at: (**a**) 2*d* below the CLC layer, (**b**) *d* below the CLC layer, (**c**) midplane of CLC layer, (**d**) *d* above the CLC layer, (**e**) 2*d* above the CLC layer. *d* - cell gap thickness, *p*_0_ = 26.5 µm, *C* = 0.84. Images are taken using a microscope objective with numerical aperture NA = 0.32.

In the naturally occurring BD texture, such as the electric-field-induced BD texture, the absolute majority of BD torons with very rare exceptions have identical appearance under the polarizing optical microscope (POM). This fact suggests that a CLC toron with T3-1 type of topological structure has the largest probability to be formed in a random process such as hydrodynamic instability. The POM appearance of BD torons depends slightly on the focusing of microscope camera, especially for BD torons of a large size, as shown in Fig. 3. As the microscope camera is focused from below to above of the CLC layer along the normal of the cell the BD torons can appear with two bright crescent points positioned at 45° with respect to crossed polarizers.

The CLC confined in a cell with homeotropic anchoring conditions has a bistability between – depending on the confinement ratio – the unwound HO or the FP textures and the BD texture. Without applied electric field the BD texture is metastable in a narrow range of confinement ratios. If the confinement ratio is too small, the anchoring force overcomes the elastic energy and unwinds torons into a uniform HO texture. If the confinement ratio is too large the BD torons compete with spontaneously forming FP texture, and are difficult or impossible to obtain. The larger the confinement ratio, the smaller is the area fraction of the BD texture. Here we define the upper limit of the confinement ratio range of a metastable BD texture as the confinement ratio at which the area fraction of the FP texture exceeds the BD texture.

**Figure 4:**
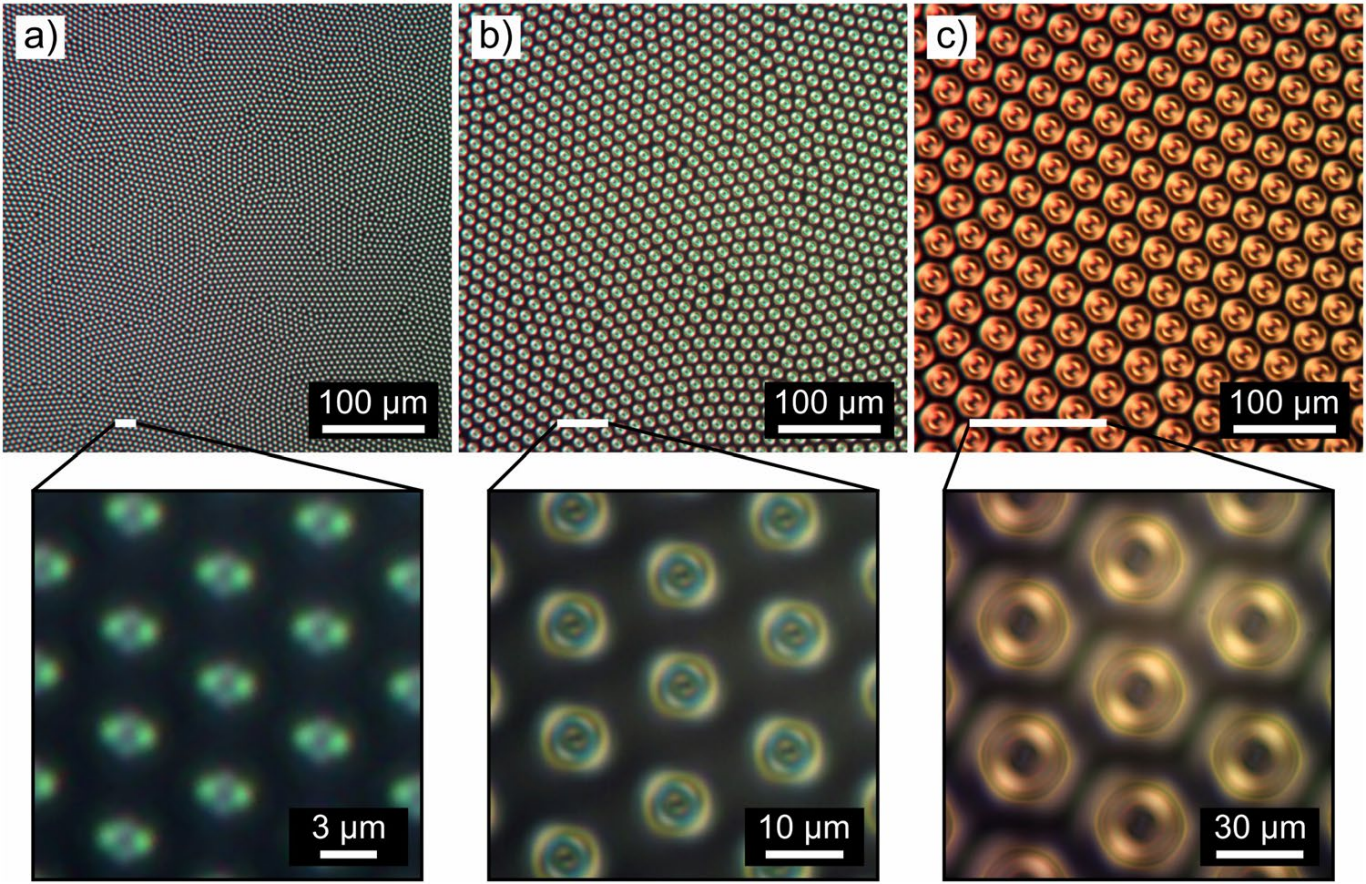
POM images of densely packed BD texture in cells with cell gaps of: (**a**) 2.9 µm, (**b**) 9.5 µm, (**c**) 22.3 µm.

**Figure 5:**
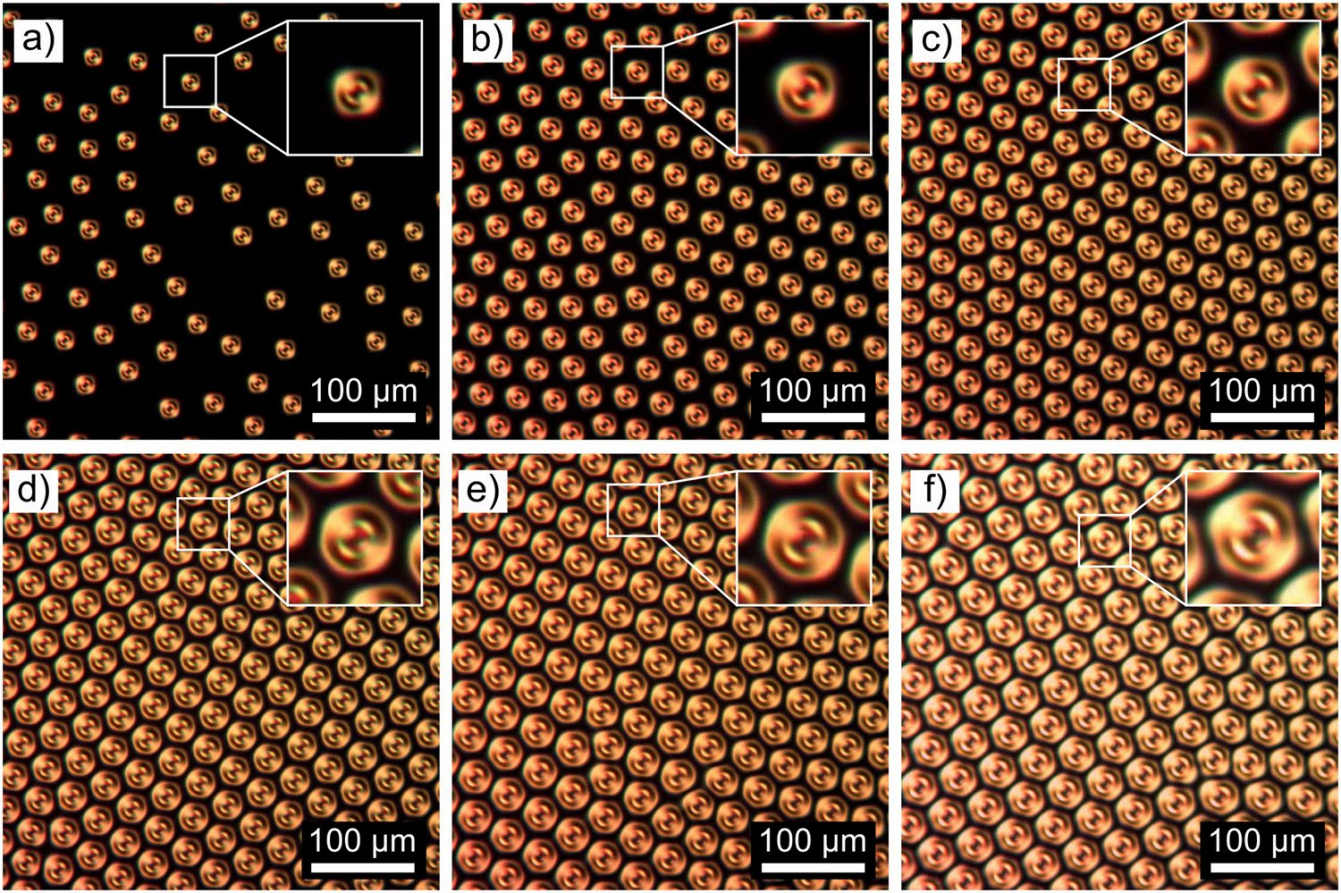
POM images showing the packing density of BD torons with *p*_0_ = 26.5 µm at different confinement ratios of the sample: (**a**) *C* = 0.64, (**b**) *C* = 0.69, (**c**) *C* = 0.73, (**d**) *C* = 0.79, (**e**) *C* = 0.84, (**f**) *C* = 0.98. Insets show images of the size of 50 × 50 µm.

The size of BD torons is proportional to the equilibrium CLC pitch and at the same time to the confinement ratio. Figure 4 shows POM images of densely packed BD texture in cells with cell gaps of 2.9 µm, 9.5 µm and 22.3 µm. For constant equilibrium CLC pitch, the maximum density of BD torons which is possible to be generated by applied electric field is proportional to the confinement ratio of the sample. Figure 5 shows POM micrographs of BD texture with confinement ratios from *C* = 0.64 to *C* = 0.98 and equilibrium CLC pitch of 26.5 µm.

In the experiments, we found the confinement ratio boundaries of the metastable region of the BD texture as well as the critical confinement ratio to be slightly dependent on the equilibrium CLC pitch or the cell gap thickness. Due to a change of the CLC pitch from 3.2 μm to 26.5 μm the lower edge of the metastable BD region increases from *C* = 0.60 to *C* = 0.67, the upper edge decreases from *C* = 1.37 to *C* = 1.19, and the critical confinement ratio *C*_*c*_ increases from 0.75 to 0.87. This indicates that the surface anchoring contribution to the balance between elastic and anchoring forces is stronger when the cell gap is smaller. The exact values of confinement ratio boundaries of the metastable BD texture are expected to depend on the elastic properties of the CLC mixture and the magnitude of anchoring energy of the CLC molecules on the alignment layer, similarly to the fact that some metastable excitations of the CLC director confined between substrates with homeotropic alignment can exist only with specific surface anchoring conditions^24^.

**Figure 6:**
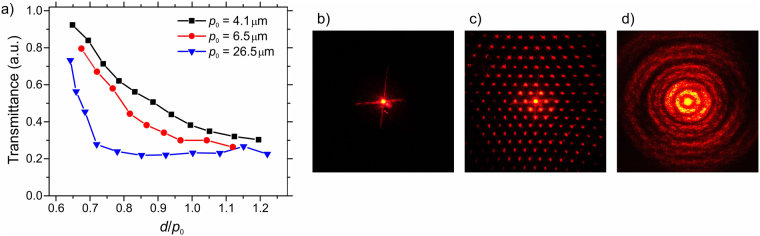
(**a**) Normalized transmittance (a.u.) of the BD texture as a function of the confinement ratio for equilibrium CLC pitch of *p*_0_ = 4.1 µm, 6.5 µm, 26.5 µm, diffraction patterns of (**b**) uniform HO, (**c**) well-aligned BD, and (**d**) not aligned BD textures.

On a macroscopic scale, the diffractive BD texture scatters light passing through it. The density of BD torons in the CLC layer defines the diffraction efficiency or optical density of the BD texture and depends on the confinement ratio. Transmittance of the BD texture at 633 nm as a function of the confinement ratio, reflecting the amount of diffracted and scattered light, is shown in Fig. 6a. Transmittance is measured as intensity of light at the point of the zero-order diffraction maximum and normalized to the transmittance of a sample with the HO texture. The CLC layer with a uniform HO texture transmits an unchanged incident laser beam to the point of zero-order diffraction maximum, Fig. 6b. At small *C*, the CLC layer contains few BD torons and the sample is weakly scattering. As *C* grows, the amount of BD torons increases and CLC scatters more light. A densely packed and well-ordered BD texture creates a distinctive two dimensional diffraction pattern consisting of a lattice of diffraction peaks reflecting the periodic alignment of torons in the CLC layer, as shown in Fig. 6c. A less ordered BD texture diffracts light into a pattern of concentric rings, as shown in Fig. 6d. Diffraction efficiency of the BD texture depends on the thickness of the CLC layer, and size and packing density of BD torons. Larger torons of a thicker CLC layer provide longer optical path for the light and create a stronger optical effect. Therefore, at the same confinement ratio a thicker sample with larger BD torons has larger diffraction efficiency compared to a thinner sample with smaller pitch and size of BD torons.

**Figure 7:**
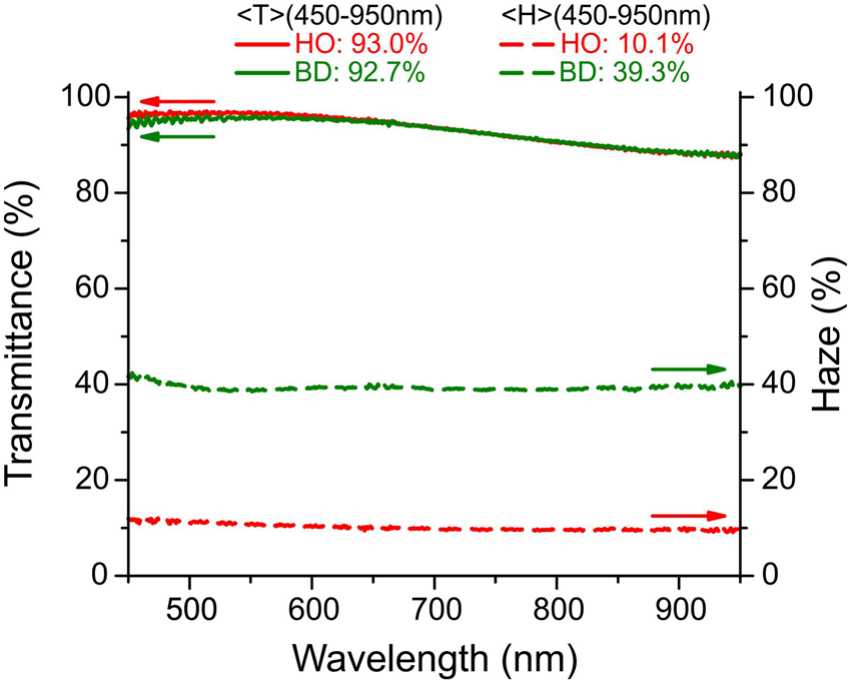
Total luminous transmittance and transmission haze of the sample with HO and BD textures as a function of wavelength. *d* = 7.0 µm, *C* = 0.68.

Switchable light scattering of the sample is investigated using spectrophotometer equipped with an integrating sphere allowing diffuse illumination of the sample with unidirectional 0.05 rad viewing. The LC cell is highly transparent in both HO and BD states, as shown in Fig. 7. The average total luminous transmittance of the sample in the range of wavelength from 450 nm to 950 nm is 93.0% and 92.7% for HO and BD textures, respectively. However, diffractive BD texture creates a significant amount of transmission haze making the LC layer act as a diffuser. The average transmission haze of the 7.0 µm sample cell with the HO texture is 10.1% compared to 39.3% with the BD texture. The amplitude of light modulation in the BD state can be controlled by changing the thickness of the LC layer and the density of BD torons.

## Conclusions

In conclusion, we have explicitly demonstrated the LC director structure of naturally formed bistable BD torons of geometrically frustrated CLCs which are generated and reliably switchable by an electric-field-induced hydrodynamic turbulence process. The topological point defects identified with a FCPM 3D LC director field reconstruction confirm that BD torons belong to the T3-1 toron type of excitations of CLC director, which are metastable due to geometrically frustrating confinement. Diffraction efficiency of densely packed BD torons are demonstrated to be proportional to the packing density, the size of torons, and the confinement ratio, reaching the maximum close to the critical value of the confinement ratio. The demonstrated fundamental topological and macroscopic optical properties of the BD texture open up new avenues for potential applications of the naturally occurring BD texture in switchable and reconfigurable diffraction gratings, highly transparent diffusers, light extracting devices, spatial light modulators and energy saving windows.

## Methods

Experiments were carried out in electro-optic LC cells fabricated from commercial glass plates coated with a transparent indium tin oxide (ITO) conductive layer. For the electrically switched cells, the inner surfaces of cells were additionally coated with the SE1211 polyimide (from HD MicroSystems) and with a 0.1 wt.% solution of CTAB surfactant (cetyl trimethyl amonium bromide, from Sigma-Aldrich) in isopropanol for homeotropic alignment. The cells for the FCPM experiments were prepared by treating the glass with a DMOAP silane (Dimethyloctadecyl[3-(trimethoxysilyl)propyl]ammonium chloride). The cell gap thickness was set using particle spacers and verified after assembly of the cell by the interference method with an Ocean Optics S2000 spectrometer.

For the purpose of the FCPM imaging experiment a low birefringence (Δn = 0.03) CLC mixture was used containing a 1:1 weight ratio of 4′-butyl-4-heptyl-bicyclohexyl-4-carbonitrile (CCN-47) and 4,4′-dipentyl-bicyclohexyl-4-carbonitrile (CCN-55) doped with 1.2 wt.% of S-811 and a small amount of BTBP dye which aligns with the LC director. The low birefringence of the LC mixture is required for mitigation of undesired lensing and polarization artefacts during FCPM imaging^47^. The HTP of S-811 in the CCN-based mixture is 8.7 μm^−1^. The cell gap thickness was 7.8 μm (measured from the confocal images) and the equilibrium CLC pitch of the mixture was 9.6 μm. The sample was imaged in 3D by conducting *xyz* scans on a Leica TCS SP5 X confocal microscope. The BTBP dye was excited with linearly polarized laser light with a wavelength of 488 nm and the fluorescence was collected in the wavelength range 515–575 nm at the same polarization. The scan was repeated at four linear polarizations separated by 45° and the director structure of a BD toron was reconstructed from the fluorescence intensities using a simulated annealing algorithm^41^. The microscopy images were taken with an effective sampling resolution of 165 nm, and the estimated spatial resolution of the method is 300 nm in the *xy* direction and approximately two times larger in the *z* direction. To estimate the angular resolution of the FCPM reconstruction method, we performed additional relaxation of the director field with a Landau-de Gennes model. We estimated how far from local elastic equilibrium each reconstructed voxel is, by relaxing the director in a 3 × 3 × 3 μm^3^ box centered on a chosen voxel, with the director around the box fixed and comparing the reconstructed and relaxed orientation. The typical deviations between the reconstructed and relaxed values of both the azimuthal and polar angle are smaller than ±5°, with the worst case angular resolution in parts of the structure being ±10° for the azimuthal angle and ±20° for the polar angle.

The CLC mixture for electro-optic experiments was prepared by adding a small amount of non-mesogenic chiral dopant CB15 (from Merck) into a negative dielectric anisotropy NLC host ZLI-4788 (from Merck; Δ*ε* = −5.7, *ε*_o_ = 10.2 at 1 kHz). The birefringence of the ZLI-4788 is Δn = 0.1647. The helical twisting power (HTP) of the chiral dopant CB15 in the ZLI-4788 was measured using the Grandjean-Cano wedge method^51^ to be HTP(CB15) = 6.3 µm^−1^. The length of equilibrium CLC pitch was selected by controlling the concentration of the chiral dopant in the CLC mixture in order to obtain a desired confinement ratio of the sample. All electro-optic experiments and characterization experiments were carried out at room temperature (22 °C).

Total luminous transmittance and transmission haze were measured using the HP 8453 UV-visible spectrophotometer equipped with the Labsphere RSA-HP-84 diffuse reflectance and transmittance accessory, and calibrated with the SRS-99-010 Calibrated Diffuse Reflectance Standard. The Labsphere accessory has a built-in 95 mm diameter integrating sphere providing diffuse illumination of the sample with unidirectional 0.05 rad viewing.
